# Case Report: First report of spinal stenosis in Imagawa-Matsumoto syndrome: a novel *SUZ12* variant in an 11-year-old Chinese child

**DOI:** 10.3389/fgene.2026.1867197

**Published:** 2026-07-14

**Authors:** Zixi Pang, Tao Zhang, Dahui Wang, Ping Xu, Bingbing Wu, Quanli Shen, Jia Hou

**Affiliations:** 1 Clinical Research Unit, Children’s Hospital of Fudan University, National Children’s Medical Center, Shanghai, China; 2 Department of Orthopaedics, Children’s Hospital of Fudan University, National Children’s Medical Center, Shanghai, China; 3 Molecular Medical Center, Children’s Hospital of Fudan University, National Children’s Medical Center, Shanghai, China; 4 Department of Radiology, Children’s Hospital of Fudan University, National Children’s Medical Center, Shanghai, China; 5 Department of Clinical Immunology and Allergy, Children’s Hospital of Fudan University, National Children’s Medical Center, Shanghai, China

**Keywords:** Imagawa-Matsumoto syndrome, overgrowth syndrome, PRC2, spinal stenosis, SUZ12

## Abstract

**Objective:**

To expand the phenotypic and genotypic spectrum of Imagawa-Matsumoto syndrome (IMMAS) by investigating the genetic etiology and unique clinical manifestations of an overgrowth case associated with a *SUZ12* gene variant.

**Methods:**

We report a case of IMMAS in an 11-year-old Chinese girl who was admitted to our hospital due to unsteady gait and an abnormal walking pattern for 1.5 years. Trio-based whole-exome sequencing (trio-WES) was performed to identify the genetic etiology. Additionally, we conducted a systematic literature review of previously reported cases of overgrowth syndromes associated with *SUZ12* variants.

**Results:**

The patient presented with generalized overgrowth, characteristic facial features, and skeletal abnormalities consistent with IMMAS. Cervical spine magnetic resonance imaging (MRI) revealed cervical canal stenosis accompanied by signs of cervical spinal cord compression and cord edema. The patient underwent posterior cervical single-door decompression, canal expansion with internal fixation, and adhesiolysis of the spinal cord and nerve roots. Trio-WES identified a *de novo* variant in *SUZ12* (NM_015355.4, c.1783_1786del; p. Lys595Profs*18). The literature review suggested that cervical spinal stenosis in our case may represent a newly reported phenotype associated with *SUZ12* variants.

**Conclusion:**

Our findings expand the phenotypic and genotypic spectrum of IMMAS. For patients with overgrowth syndromes, spinal stenosis should be considered during clinical evaluation.

## 1 Introduction

The suppressor of zeste 12 (*SUZ12*) gene encodes a core subunit of the polycomb repressive complex 2 (PRC2), a multi-subunit complex that plays a pivotal role in gene silencing. PRC2 components include enhancer of zeste homolog 2 (*EZH2*), embryonic ectoderm development (*EED*), *SUZ12*, and either retinoblastoma-binding protein 4 or 7 (*RBBP4* or *RBBP7*) (Conway et al., 2015). Heterozygous variants in *SUZ12* are associated with Imagawa-Matsumoto syndrome (IMMAS, OMIM #618786), which is characterized by overgrowth, distinctive facial features, and intellectual disability. Compared with other PRC2-related overgrowth syndromes, such as Weaver syndrome (caused by *EZH2* variants) and Cohen-Gibson syndrome (caused by *EED* variants), IMMAS is considerably rare. Cervical spinal stenosis has previously been reported exclusively in patients with *EED* variants. To date, only 19 cases of IMMAS have been reported worldwide (Imagawa et al., 2023; Yücel et al., 2024; Simsek and Vossough, 2024). Here, we report the case of an 11-year-old Chinese girl with IMMAS harboring a novel *SUZ12* variant who also exhibited cervical spinal stenosis, a previously unreported potential feature associated with *SUZ12* variants.

## 2 Case presentation

**FIGURE 1:**
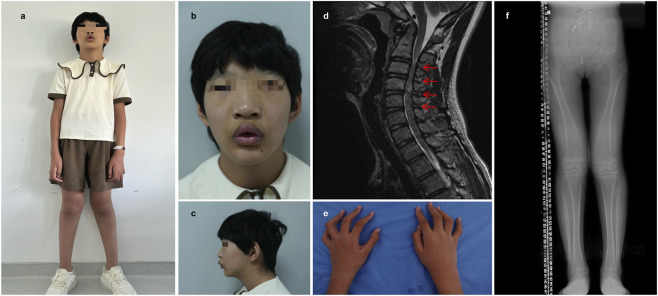
Clinical and radiological features of the patient. **(a)** Full-body photograph showing generalized overgrowth and genu valgum. **(b,c)** Frontal and lateral facial views showing characteristic facial features, including hypertelorism, a broad nasal bridge, large ears, and thick lips. **(d)** Sagittal cervical spine T2-weighted MRI showing cervical spinal canal stenosis; the AP diameter at the narrowest point was approximately 3 mm, and red arrows indicate cervical cord edema. **(e)** Photograph of both hands showing enlarged hands, elongated fingers, and flexion deformities of the distal interphalangeal joints. **(f)** Full-length standing radiograph of both lower limbs showing genu valgum. The hip-knee-ankle angle measured 4.8° on the right side and 3.7° on the left side.

An 11-year-old girl was admitted to our hospital in August 2024, with a chief complaint of abnormal gait for 1.5 years. Since the age of 10 years, the patient had experienced unsteady gait and abnormal posture characterized by a wide-based stride and swaying movements. She also experienced difficulty with sitting and standing as well as dizziness without notable triggers. Cranial magnetic resonance imaging (MRI) and echocardiography performed at a local hospital showed normal results. Over the next 8 months, her condition gradually worsened, with increasing unsteadiness, more pronounced swaying while walking, and occasional falls. At 10 years and 4 months of age (October 2023), she became unable to walk independently and developed urinary incontinence. Subsequent cranial and cervical MRI revealed cervical canal stenosis with intervertebral disc herniations at levels C2-C3, C3-C4, C4-C5, C5-C6, and C6-C7, along with signs of cervical spinal cord compression and abnormal signal intensity within the cervical spinal cord, suggestive of cord edema (Figure 1d). At 10 years and 5 months of age (November 2023), cervical computed tomography (CT) further confirmed intervertebral disc herniation at C2-C3, C3-C4, C4-C5, and C5-C6, along with narrowing of the spinal canal. The patient subsequently underwent posterior cervical single-door decompression and canal expansion with internal fixation, as well as adhesiolysis of the spinal nerve roots. However, her symptoms did not significantly improve postoperatively. She continued to exhibit gait instability and experienced sensory disturbances in her right hand. She was subsequently admitted to our hospital in August 2024.

Physical examination revealed generalized overgrowth, with a current height of 166 cm (+2.5 SD) and weight of 50.1 kg (+1.5 SD), along with facial and limb abnormalities. The patient exhibited hypertelorism, a flat nasal bridge, large ears, and thick lips. Her hands and feet were enlarged, with elongated fingers and flexion deformities of the distal interphalangeal joints (Figure 1e). Pubertal development showed Tanner stage III for breast development and Tanner stage II for pubic hair, which was appropriate for her age. Muscle tone in the lower limbs was elevated, and motion of the hip and ankle joints was restricted. Tendon reflexes were hyperactive, and ankle clonus was present.

The medical history of the patient was reviewed in detail. During the fetal period, her body length was longer than average from 12 weeks of gestation. After birth, her height consistently exceeded the average by 5–10 cm. She also exhibited poor dental development, including retention of deciduous teeth and absence of molars due to anodontia. Strabismus was identified at 3 months of age, and strabismus surgery was performed at 6 years of age. The patient showed delays in motor, cognitive, and language development, with developmental milestones lagging behind those of her peers, particularly in balance, fine motor skills, and gross motor function after 1.5 years of age. By the age of 2 years, intellectual development was also delayed.

**FIGURE 2:**
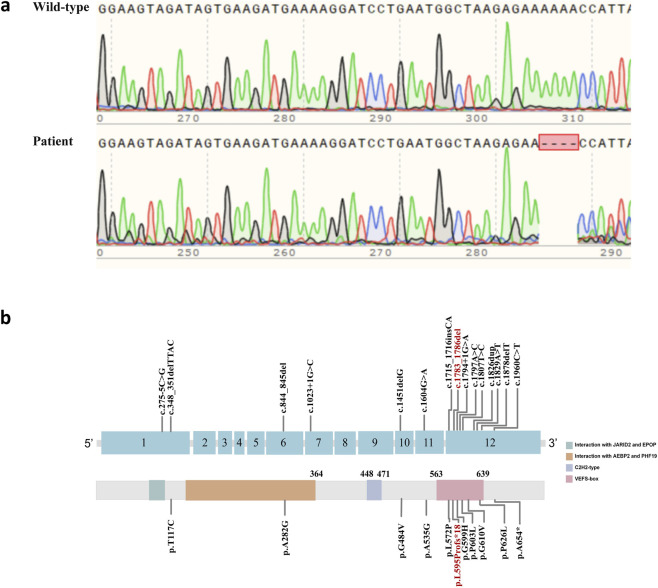
**(a)** Representative Sanger sequencing chromatograms showing the wild-type sequence and heterozygous *SUZ12* frameshift variant in the patient. The patient carried a four-base deletion, NM_015355.4:c.1783_1786del, indicated with the red box. **(b)** Schematic diagram of the *SUZ12* gene and protein structure showing the locations of reported *SUZ12* variants, as well as the variant identified in the present case. Exons 1–12 are shown in the upper panel and protein domains are shown in the lower panel. The present variant, p. Lys595ProfsTer18, is highlighted in red. Functional regions include the *JARID2*/*EPOP*-interacting region, *AEBP2*/*PHF19*-interacting region, C2H2-type zinc finger domain, and VEFS-box domain.

Based on the characteristic phenotypes of facial features, overgrowth, and developmental delay, a comprehensive clinical evaluation was performed, including assessment of the nervous, skeletal, endocrine, and psychological systems. To clarify the genetic etiology of her condition and after obtaining parental consent, whole-exome sequencing of the patient and her parents was performed using peripheral blood samples. The results revealed a heterozygous *de novo* variant in the *SUZ12* gene (NM_015355.4, c.1783_1786del; p. Lys595Profs*18), which was classified as likely pathogenic (PVS1_Strong + PS2_Moderate + PM2_Supporting) based on the American College of Medical Genetics and Genomics criteria. Sanger sequencing of the proband and both parents using peripheral blood-derived genomic DNA confirmed the frameshift *SUZ12* variant (c.1783_1786del; hg19 chr17:30322770-30322773 delAAAA) in the proband, which was absent in both parents (Figure 2).

Neurological evaluation through cervical MRI demonstrated severe cervical spinal canal stenosis, with a minimum AP canal diameter of 3 mm. Associated T2WI hyperintensity within the cervical cord suggested cord edema. No evidence of myelomalacia was observed. Pituitary MRI revealed a Rathke cleft cyst accompanied by frontal bone plate thickening. Electroencephalography indicated abnormal brain activity, with generalized slowing of electrical activity in the posterior hemispheres, predominantly consisting of theta waves at 6–7 Hz. Evoked potential testing revealed increased theta and delta wave power. Electromyography revealed neurogenic injury affecting the upper- and lower-limb muscles, indicating possible motor axonal or anterior horn cell damage.

Skeletal radiography indicated a bone age of approximately 12 years, showing mild advancement of approximately 1 year over her chronological age of 11 years, which is consistent with the overgrowth phenotype. A short, thickened left fifth metacarpal was also noted. Lumbar scoliosis was suspected, along with genu valgum and deformities of the long bones of the limbs. The hip-knee-ankle (HKA) angle for knee valgus was 4.8° on the right side and 3.7° on the left side, as shown in Figure 1f. Serum levels of growth, gonadal, and thyroid hormones were within normal ranges. Furthermore, the insulin-like growth factor 1 level was 199 ug/L, which is within the normal range for her age. The metabolic evaluation, including fasting glucose, insulin, HbA1c, liver and kidney function, and lipid profile, was normal, as were serum immunoglobulin and lymphocyte subset levels. Finally, antinuclear antibody testing was negative.

A multidisciplinary discussion was conducted with pediatric specialists in orthopedics, neurology, rehabilitation, and clinical genetics. To improve lower limb mobility and correct adductor muscle tightness, equinus deformity secondary to Achilles tendon contracture, and knee valgus, the patient underwent adductor release, gastrocnemius aponeurosis release, and epiphyseal fixation at 11 years of age. During outpatient follow-up, the patient continued to undergo physical rehabilitation twice weekly. At the latest follow-up in June 2026, her clinical condition was stable, with no further deterioration in gait stability.

## 3 Discussion

**FIGURE 3:**
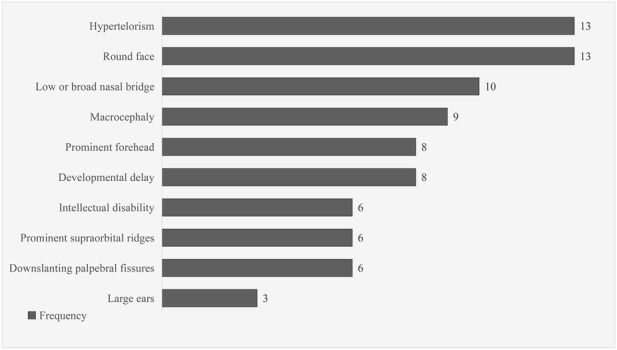
Clinical features of 19 reported patients with *SUZ12* variants.

Imagawa et al. (Imagawa et al., 2017) first described a case involving a *SUZ12* variant in 2017 in a female patient. We conducted a literature search in the Medline, PubMed, and Web of Science databases using the search terms “Imagawa-Matsumoto syndrome” or “IMMAS” or “overgrowth,” and “*SUZ12*” between 2000 and 2025. Original case reports and case series describing individuals with clinically and/or genetically confirmed Imagawa-Matsumoto syndrome were included. Review articles, conference abstracts, animal studies, *in vitro* studies, duplicate reports, and publications lacking sufficient clinical or genetic information were excluded. The study selection process is summarized in Supplementary Figure S1. The identified cases were reviewed to summarize the clinical features of IMMAS. To date, only 19 cases with *SUZ12* variants have been documented (Imagawa et al., 2023; Yücel et al., 2024), with the majority reported from the United States (5/19), Japan (3/19), Turkey (3/19), and China (2/19). One case was reported based solely on MRI findings and some postpartum clinical manifestations (Simsek and Vossough, 2024). Among the 19 patients, seven (36.8%) were female, and the median age at diagnosis was 12 years (range, fetal to 41 years). Most cases were diagnosed during adolescence. Reported facial features included macrocephaly (9/18), round face (13/18), prominent forehead (8/18), hypertelorism (13/18), and a low, broad nasal bridge (10/18). In addition, varying degrees of intellectual, behavioral, and motor developmental delays were observed in 12 of the 19 patients. Musculoskeletal manifestations often included enlarged hands or feet (9/18) and contractures of the fingers or toes (11/18). Imaging findings occasionally revealed cortical malformations (2/18), corpus callosum dysgenesis (2/18), arachnoid cysts, and Chiari type I malformation (2/18). Urological abnormalities, such as cryptorchidism, were noted in four of the 12 male patients. The clinical features of reported patients with *SUZ12* variants are shown in Figure 3.

Based on the clinical presentation of the patient, differential diagnoses among other pediatric overgrowth syndromes and tall-stature disorders were considered, including Sotos syndrome (*NSD1* variants), Marfan syndrome (*FBN1* variants), and PRC2-related conditions such as Weaver (*EZH2* variants) and Cohen-Gibson (*EED* variants) syndromes. Sotos syndrome shares features such as macrocephaly and advanced bone age but typically presents with a long, triangular face and pointed chin (Baujat and Cormier-Daire, 2007). Marfan syndrome is characterized by tall stature and long fingers, but cognitive development is typically normal (Yuan and Jing, 2010). Weaver and Cohen-Gibson syndromes show overlapping intellectual disability and overgrowth; however, they are differentiated by specific facial features (such as prominent round chin in Weaver) and can be genetically confirmed through molecular genetic testing (Griffiths et al., 2019). In our patient, the identification of a *de novo* pathogenic *SUZ12* variant, together with the characteristic clinical phenotype, supported a diagnosis of Imagawa-Matsumoto syndrome.

However, spinal stenosis has not been reported in any of the 19 previously described cases. Here, we describe a patient with IMMAS harboring a novel *SUZ12* variant who also presented with cervical spinal stenosis, a feature not previously reported. Individuals with *EED* variants have been shown to exhibit cervical spine abnormalities, including spinal canal stenosis and scoliosis (Griffiths et al., 2019; Cohen and Gibson, 2016; Cohen et al., 2015; Smigiel et al., 2018). Griffiths et al. (Griffiths et al., 2019) reported a patient with an *EED* variant who was diagnosed with cervical spinal canal stenosis and had progressive gait deterioration and bilateral internal rotation of the feet at 11 years of age. Cooney et al. (Cooney et al., 2017) reported cervical spine stenosis in a patient with *EED*-related Weaver syndrome. Because *SUZ12* and *EED* are interacting subunits of the PRC2 complex, insights may be drawn from *EED*-related overgrowth syndromes. Although cervical spinal stenosis has been reported in individuals with *EED* variants, whether similar mechanisms contribute to the spinal phenotype observed in our patient remains unknown. Further studies are required to clarify the potential role of *SUZ12* variants in the development of spinal abnormalities.

PRC2, composed of the core subunits *SUZ12*, *EED*, and *EZH2*, catalyzes histone H3 lysine 27 trimethylation (H3K27me3) to silence target genes epigenetically. *SUZ12* serves as a scaffolding protein with four defined domains, including the VEFS-box domain affected in our patient, as shown in Figure 3. The identified variant, p. Lys595Profs*18, is located within this evolutionarily conserved domain (spanning from *Drosophila* to humans (Cyrus et al., 2019; Sharaf et al., 2022)). Notably, although cervical spinal stenosis has been reported in individuals with *EED* variants involving the WD4/WD5 beta-propeller domains, whether similar PRC2-related mechanisms contribute to the spinal phenotype observed in our patient remains unknown. Because of the functional interplay among PRC2 core components, disruption of the *SUZ12* VEFS-box domain may affect pathways relevant to skeletal development; however, this hypothesis currently lacks direct functional evidence and requires further investigation.

Genome-wide studies have identified polycomb-regulated genes involved in skeletal and bone differentiation (Bracken et al., 2006). Specifically, *EZH2* represses osteogenesis by trimethylating H3K27me3 at the promoters of key genes such as *RUNX2,* a transcription factor pivotal for osteoblast differentiation (Komori, 2019). Notably, *SUZ12*-knockout embryonic stem cells exhibit significantly reduced *EZH2* binding and H3K27me3 signaling, indicating that *SUZ12* initiates PRC2 assembly and functions as a prerequisite for *de novo* H3K27me3 establishment (Højfeldt et al., 2018). Although these findings support a role for PRC2 in skeletal development, their relevance to cervical spinal stenosis in IMMAS remains to be established.

In two previously reported patients with spinal stenosis due to *EED* variants, no therapeutic interventions were described. Our patient underwent surgery due to the significant impact of the condition on her daily life and physical activity. However, the long-term prognosis remains uncertain and requires continuous follow-up. To the best of our knowledge, no current literature clearly outlines specific treatments for IMMAS. Most management strategies rely on symptomatic treatment and regular evaluations, with only one publication addressing the follow-up of children with IMMAS (Yüksel Ülker et al., 2023). This includes regular assessments of muscle and skeletal health, management of joint pain and scoliosis, and referrals for physical therapy and orthopedic surgery when necessary. In addition, the learning and behavioral abilities of patients should be evaluated periodically, and appropriate speech therapy should be provided. Although no tumors have been reported in children with *SUZ12* variants, *SUZ12* dysfunction has been associated with the development of squamous cell carcinoma of the head and neck. Moreover, neuroblastoma and acute lymphoblastic leukemia have been observed in children with Weaver syndrome (associated with *EZH2* variants). Therefore, close monitoring of tumor development in children with *SUZ12* variants is essential during follow-up. Collectively, these findings indicate that spinal stenosis may represent a clinically relevant feature requiring attention in patients with *SUZ12* variants.

## 4 Conclusion

Our study expands the phenotypic and genotypic spectrum of IMMAS by identifying spinal stenosis as a novel potential feature in patients with *SUZ12* variants. Although the causal relationship and precise biological links remain to be functionally validated, our findings emphasize the importance of genetic testing and proactive spinal surveillance in these patients. Future research should focus on elucidating the underlying molecular mechanisms and exploring targeted therapies to improve the clinical outcomes of patients with IMMAS.

## Data Availability

The datasets presented in this article are not readily available because they contain potentially identifiable clinical, imaging, and genetic information from a minor and are subject to ethical and privacy restrictions. Requests to access the datasets should be directed to the corresponding author.

## Supplementary material

SUPPLEMENTARY FIGURE S1 Flow diagram of the literature search and study selection process.
