# Supplementary material for: Case Report: First report of spinal stenosis in Imagawa-Matsumoto syndrome: a novel SUZ12 variant in an 11-year-old Chinese child
Source: Front Genet. 2026 Jul 14;17:1867197. doi: 10.3389/fgene.2026.1867197 (PMC13406684; doi:10.3389/fgene.2026.1867197)
Supplement: Supplementary file 1 [file Table1.docx]

**Supplementary table 1. Clinical and genetic features in 20 IMMAS individuals with *SUZ12* variants.**

| **Patient No.** | **Age/**  **Gender** | **Country** | **Nucleotide change** | **Amino acid change** | **Inheritance** | **Facial Characters** | **Growth and Development** | **Musculoskeletal System** | **Nervous System** | **Urogenital System** | **Other** |
| --- | --- | --- | --- | --- | --- | --- | --- | --- | --- | --- | --- |
| 1^[1]^ | 11y/F | Japan | NM_015355.4:c.1829A>T | p.Glu610Val | paternal | round face, macrocephaly, broad forehead, flat occiput, hypertelorism, large ears | moderate intellectual disability | flexion disorder of fingers, camptodactyly (mild), ingrown nails of halluces, short 2nd and 4th toes (bilateral), large hands and feet, mild scoliosis, plantar skin defects, knee joints contracture (mild), atrophy of gastrocnemius muscles | arachnoid  cysts, chiari  malformation type I | NA | NA |
| 2^[1]^ | 41y/M | Japan | NM_015355.4:c.1829A>T | p.Glu610Val | NA | round face, broad forehead, hypertelorism, prominent chin crease | NA | large hands and feet | NA | NA | NA |
| 3^[2]^ | 19y/M | Brazil | NM_015355.4:c.1797A>C | p.Gln599His | NA | round face, macrocephaly, hypertelorism, prominent  and/or long philtrum, large ears, downslanting palpebral  fissures | NA | broad metaphyses, cubitus valgus, clinodactyly of bilateral first, second and fifth toes | NA | NA | NA |
| 4^[2]^ | 9y/F | France | NM_015355.4:c.844_845del | p.Ala282Glnfs*7 | De novo | round face | NA | short fifth fingers, clinodactyly and ungual hypoplasia of fifth toes | non-febrile seizure | NA | hypermetropia,  Strabismus,  Chronic constipation |
| 5^[2]^ | 11y/F | Japan | NM_015355.4:c.1829A>T | p.Glu610Val | paternal | round face, macrocephaly, hypertelorism, large ears | moderate intellectual  disability | broad metaphyses, flexion disorder of fingers, camptodactyly (mild), ingrown nails of halluces, short second and fourth toes (bilateral), mild scoliosis, plantar skin defects, knee joints contracture (mild), atrophy of gastrocnemius muscles | metaphyses, hypotonia, enlarged lateral and third ventricles, arachnoid cysts, Chiari malformation type I | NA | NA |
| 6^[3]^ | 11y/F | USA | NM_015355.4:c.348_351delTTAC | p.Tyr117 CysfsTer24 | NA | round face, supraorbital and ridges prominent, hypertelorism, broad and low nasal bridge,prominent chin | developmental delay, borderline Intellectual disability | bilateral short fifth fingers | NA | NA | NA |
| 7^[3]^ | 11y/F | USA | NM_015355.4:c.1604G>A | p.Arg535Gln | NA | broad and low nasal bridge | developmental delay; severe-to-profound intellectual disability | pes planus, bilateral coxa valga deformity | periventricular leukomalacia, hypotonia | NA | esotropia/exotropia,Mild persistent  ashma |
| 8^[3]^ | 11y/F | Portugal | NM_015355.4:c.1715_1716insCA | p.Leu572 PhefsTer11 | De novo | triangular face, forehead or increased bifrontal diameter prominent, telecanthus, downslanting palpebral fissures,broad and low nasal bridge | developmental  delay, mild Intellectual disability | hypotonia, large hands and feet, camptodactyly or clinodactyly finger and toe | NA | NA | NA |
| 9^[3]^ | 2.5y/M | Finland | NM_015355.4:c.1807T>C | p.Phe603Leu | Paternal | round face, macrocephaly and head shape,forehead or increased bifrontal diameter prominent, hypertelorism, broad and low nasal bridge | developmental delay | hypotonia, large hands and feet, camptodactyly or clinodactyly toe | NA | cryptorchidism | excessive loose skin, small toenails, frequent infections |
| 10^[3]^ | 27y/M | Finland | NM_015355.4:c.1807T>C | p.Phe603Leu | Maternal | round face, macrocephaly and head shape, brachycephaly, forehead or increased bifrontal diameter prominent, prominent supraorbital ridges, hypertelorism, downslanting palpebral fissures, everted lower lid, broad and low  nasal bridge, prominent chin/jaw | NA | pectus, large hands and feet, camptodactyly or clinodactyly toe, bilateral foot pronation, pes planus, mild hypermobility of joints | NA | cryptorchidism | atypically soft skin, frequent infections |
| 11^[3]^ | 18y/M | China | NM_015355.4:c.1878delT | p.Phe626 LeufsTer7 | De novo | macrocephaly and head shape, hypertelorism, broad and low nasal bridge, large hands | NA | NA | NA | NA | NA |
| 12^[3]^ | 7.5y/M | Canada | NM_015355.4:c.1960C>T | p.Arg654Ter | De novo | round face, prominent supraorbital ridges, hypertelorism, long palpebral fissures, broad and low nasal bridge | developmental delay, mild autism, intellectual  disability | hypotonia, with “barrel-shaped” and asymmetrical chest, camptodactyly or clinodactyly finger, narrow and “rounded” shoulders, short clavicles, scapular winging, decreased muscle bulk, mildly hypermobile joints prominent digit pads on toes | corpus callosum hypoplastic |  | patent ductus arteriosus, patent foramen ovale |
| 13^[3]^ | 19y/M | USA | NA | NA | maternal | macrocephaly and head shape, brachycephaly, prominent forehead or increased bifrontal diameter, prominent supraorbital ridges, downslanting palpebral fissures, broad and low nasal bridge | developmental delay, mild autism, intellectual disability | large hands and feet, bilateral short fourth fingers | NA | cryptorchidism | NA |
| 14^[3]^ | 15.5y/M | USA | NA | NA | De novo | macrocephaly and head shape, prominent supraorbital ridges, enlarged frontal sinuses, hypertelorism, downslanting palpebral fissures | developmental  delay, intellectual disability, learning disabilities | large hands and feet, finger camptodactyly or clinodactyly | complete agenesis corpus callosum | cryptorchidism, disjoined epididymides | NA |
| 15^[4]^ | Infancy/M | Turkey | NA | NA | NA | round face, prominent forehead, hypertelorism, epicanthal fold, micrognathia, and full cheeks, an elongated face and beak-shaped nose developed | severe motor and mental retardation | contractures in the elbow and knee, and camptodactyly | NA | NA | NA |
| 16^[4]^ | Infancy/M | Turkey | NA | NA | NA | round face, prominent forehead, hypertelorism, epicanthal fold, micrognathia, and full cheeks | severe motor and mental retardation | NA | NA | NA | NA |
| 17^[5]^ | 19y/F | Turkey | NA | NA | De novo | round face, prominent forehead, broad nasal ridge | NA | flat feet, large hands, camptodactyly, and clinodactyly | agenesis of the corpus callosum and polymicrogyria | irregular menstrual periods, ctopically located left kidney in the pelvis | NA |
| 18^[6]^ | Fetal/M | U.S.A | NM_015355.4:c.1451delG | NA | NA | NA | NA | NA | cortical malformation with abnormal perirolandic gyri and polymicrogyria | anteriorly placed anus | NA |
| 19^[7]^ | 12y/M | Korea | 17q11.2 deletion, chr17:29,935,893-31,361,994, 1.4 Mb, GRCh37/hg19 | Whole gene deletion | De novo | round face, macrocephaly, prominent forehead, hypertelorism, downslanting palpebral fissures, low or broad nasal bridge | developmental delay, intellectual disability | large hands and feet, cavovarus foot deformity,  hypermobility of wrist  and finger joints | NA | NA | excessive loose skin |
| 20  our case | 11y/F | China | NM_015355.4:c.1783_1786del | p.Lys595ProfsTer18 | De novo | macrocephaly and head shape, prominent supraorbital ridges, broad and low nasal bridge, hypertelorism,large and thick lips | generalized overgrowth; tall stature since fetal life; height 166 cm (+2.5 SD), weight 50.1 kg (+1.5 SD); mild advanced bone age; motor, cognitive, language, and intellectual developmental delay; age-appropriate puberty. | large hands and feet; elongated fingers; distal finger flexion; mild lower-limb hypertonia; limited hip and ankle range of motion; genu valgum; cervical disc herniation with canal stenosis | hyperreflexia; positive ankle clonus; left temporal encephalomalacia-like lesion; cranial bone plate thickening; Rathke’s cleft cyst | NA | strabismus |

### **Abbreviations:** **NA:** Not applicable; **IMMAS:** Imagawa-Matsumoto syndrome;**F**：Female; **M**: Male

**Reference**

[1] IMAGAWA E, HIGASHIMOTO K, SAKAI Y, et al. Mutations in genes encoding polycomb repressive complex 2 subunits cause Weaver syndrome [J]. Hum Mutat, 2017, 38(6): 637-48.

[2] IMAGAWA E, ALBUQUERQUE E V A, ISIDOR B, et al. Novel SUZ12 mutations in Weaver-like syndrome [J]. Clin Genet, 2018, 94(5): 461-6.

[3] CYRUS S S, COHEN A S A, AGBAHOVBE R, et al. Rare SUZ12 variants commonly cause an overgrowth phenotype [J]. Am J Med Genet C Semin Med Genet, 2019, 181(4): 532-47.

[4] YüKSEL ÜLKER A, ULUDAĞ ALKAYA D, ÇAĞLAYAN A O, et al. An investigation of the etiology and follow-up findings in 35 children with overgrowth syndromes, including biallelic SUZ12 variant [J]. Am J Med Genet A, 2023, 191(6): 1530-45.

[5] YüCEL Z, YüKSEL E B, KOç A. Imagawa-Matsumoto Syndrome: The First Case From Turkey [J]. Noro Psikiyatr Ars, 2024, 67(3): 289-92.

[6] SIMSEK O, VOSSOUGH A. Fetal and postnatal neuroimaging of SUZ12-related overgrowth: Imagawa-matsumoto syndrome [J]. J Neuroradiol, 2024, 51(5): 101210.

[7] Park S, JANG MA. Identification of SUZ12 Haploinsufficiency due to a 1.4-Mb Deletion at 17q11.2 in a Child With Overgrowth and Intellectual Disability Syndrome[J]. Ann Lab Med. 2023,43(3):319-322.
