# Supplementary figures and images for: Case Report: First report of spinal stenosis in Imagawa-Matsumoto syndrome: a novel SUZ12 variant in an 11-year-old Chinese child
Source: Front Genet. 2026 Jul 14;17:1867197. doi: 10.3389/fgene.2026.1867197 (PMC13406684; doi:10.3389/fgene.2026.1867197)

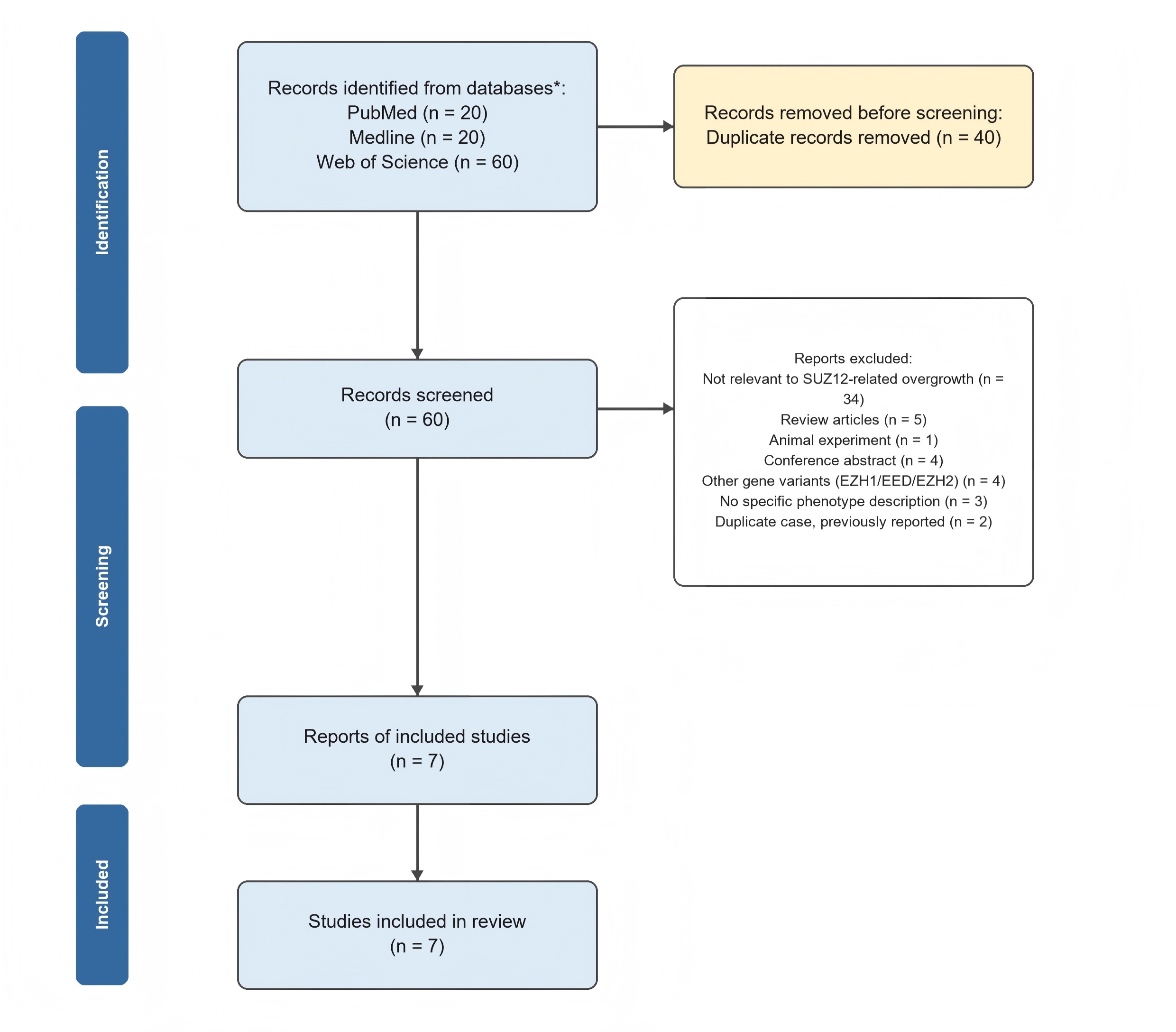

Supplement: Supplementary file 2 [file Image1.jpg]
